# Supplementary material for: Chromothripsis during telomere crisis is independent of NHEJ, and consistent with a replicative origin
Source: Genome Res. 2019 May;29(5):737–49. doi: 10.1101/gr.240705.118 (PMC6499312; doi:10.1101/gr.240705.118)
Supplement: Supplemental Material [file supp_gr.240705.118_Supplemental_file_1.zip › contigs/annotated_contigs/DB116/contig.2.DB116_length_433_mean_cov_6.58198614319.docx]

**DB116_length_433_mean_cov_6.58198614319**

```
TAAACTATATTATTAAAATACGATAAAAGGAATGAAGTAAACAAACAAGGGCACATAAGTAAAAACAGATTAGCAAGGCAAGAATTTTC
 >chr13:54772513-54772738 - E=6e-121
TGAAGAGTTGCATTTAATTTGGGAACAGAAAGCTTGAGGTATTATGAAAGAGAATTATACAATTTGAAATTTAAGAGTGAGTCATGGGC

TAGATTATCCAGGCCTTTGCTGAGCTTGGAAGTTTGGCTTT|TATCTG|TCTCCTTAGATCAACGTAAAAAGCAATAATGGGACTGTGC
                                          >chr13:54771073-54771287 - E=3e-117
ATCCTTTTGAGCTTTTCTATTACCAGTTAAATTTTAAATTCCTTTTACAAATACTAAGAAAATTATATAGTCATGTGAAAAATTAATAG

AATGAACAAAGAAAAAGAAATACATATCACTTCACTGCTCTGCTTGCCTTTAAAGATATATCTGAGAGAATCATTTGGA
```
